# Modelling the complexity of heart failure with preserved ejection fraction 

**DOI:** 10.1093/cvr/cvy095

**Published:** 2018-05-08

**Authors:** Kevin O’Gallagher, Ajay M Shah

**Affiliations:** School of Cardiovascular Medicine and Sciences, James Black Centre, King's College London British Heart Foundation Centre of Excellence, 125 Coldharbour Lane, London SE5 9NU, UK

**This editorial refers to ‘Multiple common comorbidities produce left ventricular diastolic dysfunction associated with coronary microvascular dysfunction, oxidative stress, and myocardial stiffening’ by O. Sorop *et al.*, pp. 954–964.**

A substantial proportion of patients with heart failure have a left ventricular (LV) ejection fraction (EF) in the ‘normal’ range, a form of the syndrome that is termed heart failure with preserved ejection fraction (HFpEF). Patients with HFpEF have significant morbidity and mortality but unlike heart failure with reduced EF, there are currently no effective validated therapies. HFpEF is therefore an important area for further research. Patients with HFpEF have cardiac and extra-cardiac manifestations, including LV diastolic dysfunction, abnormal heart rate and rhythm, microvascular dysfunction, increased aortic stiffness, and abnormal ventriculo-vascular coupling, which impair systolic and diastolic reserve capacity upon exercise.^1^ The underlying pathophysiology is incompletely understood, in part because HFpEF is highly heterogenous and may not represent a single condition. Patients with HFpEF frequently have comorbidities such as hypertension, obesity, Type II diabetes, hyperlipidaemia, and renal disease (*Figure 1*). However, not all patients have all comorbidities and the unpredictable interplay between different comorbidities is likely to result in multiple HFpEF phenotypes. Indeed, unbiased cluster analysis of densely phenotyped HFpEF patients suggests the presence of distinct ‘phenogroups’ with different clinical characteristics and outcomes.^2^

**Figure 1:**
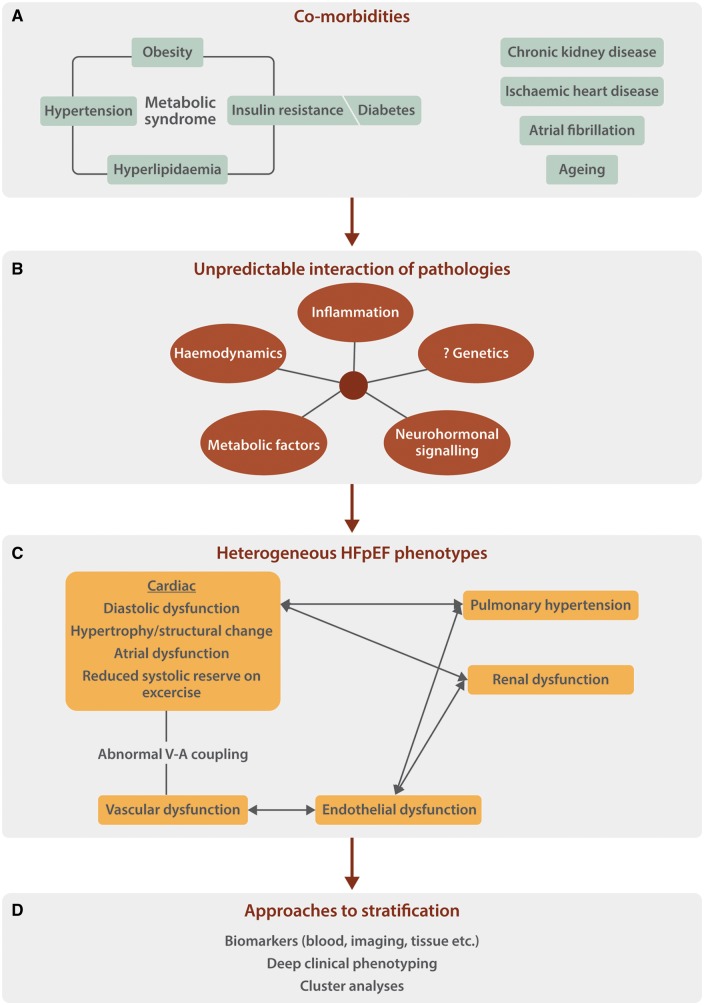
Proposed framework for the investigation of HFpEF as a heterogenous condition. The patient-specific interplay among those comorbidities that are present (*A*) will influence the pathophysiological mechanisms at play (*B*). These in turn will determine the precise pattern of the clinical phenotype (*C*). Stratification of patients with HFpEF, both to inform underlying pathophysiology and ultimately to select effective treatment, could be based on a combination of factors (*D*). The selection of animal models and interpretation of data obtained in such models would be optimized by consideration of such a framework.

Clinical studies to investigate the pathomechanisms involved in HFpEF have typically involved small numbers of highly selected patients subjected to invasive physiological assessment and cardiac biopsy-based analyses. A recent extensively-promoted model based on such studies posits that comorbidities induce systemic inflammation and vascular endothelial dysfunction as a consequence of an abnormal balance between reactive oxygen species (ROS) production and nitric oxide (NO) bioavailability.^3^ Consequent abnormalities of NO/cyclic GMP (cGMP)/protein kinase G (PKG) signalling are proposed to drive increased cardiomyocyte stiffness and interstitial cardiac fibrosis, thereby leading to LV diastolic dysfunction, as well as abnormalities in the lungs, kidneys, and skeletal muscle that contribute to clinical dysfunction. The generalisability of this hypothesis is debatable given the small sample sizes, the marked heterogeneity of clinical HFpEF, and the well-recognized limitations of endomyocardial biopsy-based analyses in patients, but it serves as a useful basis for more detailed investigation.

Experimental animal models that allow deeper analysis of the pathogenesis of HFpEF would clearly be valuable. Rodent models that have been employed include the DOCA-salt model of hypertension and models of obesity and/or diabetes.^4^ The advantage of these models is the ability to use genetic modification to investigate mechanisms but the disadvantage is the difficulty in mimicking the clinical characteristics of human HFpEF in small animals. In this issue, Sorop *et al.*^5^ report a porcine model in which a combination of comorbidities led to LV diastolic dysfunction with preserved EF. These authors used a combination of streptozotocin-induced diabetes, surgical renal intervention to induce kidney dysfunction and hypertension, and a high fat/high salt diet in a relatively small group of animals, but which were followed up for 6 months and intensively investigated. The authors were able to document key abnormalities including evidence of systemic inflammation, coronary endothelial dysfunction, an increase in ROS levels, a decrease in NO with evidence of NOS uncoupling, and an increase in cardiomyocyte passive stiffness and myocardial fibrosis.

Sorop *et al.*^5^ should be congratulated for a detailed study which will be of particular interest to those investigating the potential role of abnormal NO signalling in HFpEF. They found evidence of eNOS uncoupling in their model; i.e. a change from dimeric to monomeric eNOS which leads to superoxide instead of NO production and is implicated in diverse cardiovascular pathologies. eNOS uncoupling involves a vicious cycle in which superoxide further impairs eNOS activity both by depleting the essential co-factor tetrahydrobiopterin and inhibitory phosphorylation of eNOS at residue Y657. The authors report good evidence that a substantial proportion of myocardial superoxide originated from uncoupled NOS enzymes, based on the efficacy of the NOS inhibitor L-NAME, although data to support NADPH oxidases as an important source was less convincing. Interestingly, no significant changes were found in cGMP levels or the activities of PKG and phosphodiesterase 5 (which degrades cGMP), raising the question as to how (or if) the reduced NO bioavailability is linked to LV diastolic dysfunction (and different from the data reported in human studies^6^^,^^7^). The authors also investigated changes in the giant cytoskeletal protein, titin, which is considered to be a key determinant of passive cardiomyocyte stiffness. Titin abnormalities could be involved in HFpEF through three processes—an isoform-shift from the N2BA to N2B form, altered phosphorylation and oxidative modification—the first two of which have been found in cardiac biopsies from HFpEF patients.^6^^,^^8^ While Sorop *et al.*^5^ found increased expression of the N2B titin isoform, consistent with increased passive stiffness, no titin hypo-phosphorylation was found—which is suggested to be related to decreased NO/PKG activity and again raises the question as to the precise relationship between changes in NO signalling and diastolic dysfunction. Indeed, a significant limitation of this study is that its design did not allow mechanistic links to be established among the different abnormalities. Other limitations include the use of streptozotocin to induce diabetes, which probably does not closely mimic the human setting—as suggested by Sorop *et al.’*s findings that cardiomyocyte size and body weight were both reduced in their model rather than being increased as might be expected. With regard to inflammation, the authors measured systemic TNFα levels but it would have been informative to also quantify myocardial cytokines and inflammatory cell infiltration.

A more general critique of the model reported by Sorop *et al.*^5^ is whether the approach of combining multiple comorbidities in this manner is the best way to model HFpEF? It is highly unlikely that any single model can adequately map the pathophysiology of all HFpEF. For example, a different porcine model to induce LV diastolic dysfunction reported by Schwarzl *et al.*^9^ used a combination of DOCA-salt hypertension and a western diet, and found some different abnormalities such as significant cardiomyocyte hypertrophy and titin hypo-phosphorylation. In our opinion, a combination of approaches is required to significantly advance our knowledge of the pathogenesis of HFpEF and develop effective treatments. Phenotype-specific large animal models promise to be an important component of the armamentarium, allowing initial assessment of new therapeutic approaches targeted against specific pathways, while a more rigorous clinical phenotyping, experimental investigation and classification of patients with HFpEF is also required (*Figure 1*).

## Funding

The work was supported by the British Heart Foundation (BHF CH/1999001/11735) and the Department of Health via a National Institute for Health Research (NIHR) Biomedical Research Centre award to Guy’s & St Thomas’ NHS Foundation Trust in partnership with King’s College London and King’s College Hospital NHS Foundation Trust.

**Conflict of interest:** none declared.
